# Infectious Diseases in General Hospitals

**Published:** 1907-12-14

**Authors:** 


### Resident Medical Officers' Department.

[The Editor accepts no responsibility for any opinions expressed in these columns, which are freely open to all resident medical officers. Discussion and contributions are invited, and, if the latter are accepted, they will be paid for.]

### INFECTIOUS DISEASES IN GENERAL HOSPITALS.

With reference to the article under the above heading, which was published in this column in our issue of November 16, page 180, the Medical Superintendent of an important Scottish hospital writes to us as follows:—

"I conclude from the material in the Resident Medical Officers' Column that the communication under the above heading comes from a member of the profession on your side of the Border. He speaks of erysipelas cases being treated in medical wards, and further down he states that, 'if infectious disease supervene, the case *should be sent home*, or to the hospital for infectious diseases.' As the information in this column is intended to be a guide to the junior house physican, it should have been made perfectly clear that no case can be *sent home* unless in the ambulance waggon belonging to the Public Health Authorities. The writer, of course, must know it is quite illegal to convey a patient suffering from any infectious disease by means of a cab or other public conveyance, or even expose the public in any way to the risks of infection. Less than three months ago I was appealed to by one of my juniors. The parents of a child suffering from measles insisted on removing the child to their own home (a distance of twelve miles) either by train or in a public conveyance rather than send it to the hospital for infectious diseases. My house surgeon did not seem to be fully aware that such a procedure was entirely illegal, and when I explained the matter to the parents they at once agreed that the child should be removed to the fever hospital. I think these articles in the Resident Medical Officers' column are helpful, and if the points similar to the one I have indicated were emphasised they would be still more helpful in the future. In a general hospital where there is a Resident Medical Superintendent administrative difficulties are not so likely to occur as in small hospitals where the resident who is a junior has no one to guide him and acts entirely on his own responsibility. The method I adopt here prevents many mistakes and much irritation. In our general office there is a book called the 'Transfer Book,' and no patient can be removed from one ward to another, or from this infirmary to any fever hospital, or to any poor-house, or to an asylum, without all the particulars being first of all entered in this book, and no action is taken until it bears my signature. In this way the risk of a junior resident, through want of knowledge, allowing, for instance, an infectious case to be removed in an irregular way is avoided."

